# Context‐dependent venom deployment and protein composition in two assassin bugs

**DOI:** 10.1002/ece3.6652

**Published:** 2020-08-17

**Authors:** Maike L. Fischer, Natalie Wielsch, David G. Heckel, Andreas Vilcinskas, Heiko Vogel

**Affiliations:** ^1^ Department of Entomology Max Planck Institute for Chemical Ecology Jena Germany; ^2^ Research Group Mass Spectrometry/Proteomics Max‐Planck Institute for Chemical Ecology Jena Germany; ^3^ Institute for Insect Biotechnology Justus Liebig University Giessen Germany

**Keywords:** assassin bug, defense venom, prey‐killing venom, proteomics, transcriptomics, zoophagy

## Abstract

The Heteroptera are a diverse suborder of phytophagous, hematophagous, and zoophagous insects. The shift to zoophagy can be traced back to the transformation of salivary glands into venom glands, but the venom is used not only to kill and digest invertebrate prey but also as a defense strategy, mainly against vertebrates. In this study, we used an integrated transcriptomics and proteomics approach to compare the composition of venoms from the anterior main gland (AMG) and posterior main gland (PMG) of the reduviid bugs *Platymeris biguttatus* L. and *Psytalla horrida* Stål. In both species, the AMG and PMG secreted distinct protein mixtures with few interspecific differences. PMG venom consisted mostly of S1 proteases, redulysins, Ptu1‐like peptides, and uncharacterized proteins, whereas AMG venom contained hemolysins and cystatins. There was a remarkable difference in biological activity between the AMG and PMG venoms, with only PMG venom conferring digestive, neurotoxic, hemolytic, antibacterial, and cytotoxic effects. Proteomic analysis of venom samples revealed the context‐dependent use of AMG and PMG venom. Although both species secreted PMG venom alone to overwhelm their prey and facilitate digestion, the deployment of defensive venom was species‐dependent. *P. biguttatus* almost exclusively used PMG venom for defense, whereas *P. horrida* secreted PMG venom in response to mild harassment but AMG venom in response to more intense harassment. This intriguing context‐dependent use of defensive venom indicates that future research should focus on species‐dependent differences in venom composition and defense strategies among predatory Heteroptera.

## Graphical abstract

In true bugs, venom is used not only to kill and digest invertebrate prey but also defensively when they are disturbed. We carried out a comprehensive analysis of venom components from two assassin bug species, employing a combination of transcriptomic and proteomic approaches, conducted in vitro bioactivity assays to investigate venom effects and identified context‐dependent venom deployment. Our comprehensive analysis of venom composition, effects, and deployment by two assassin bugs revealed intriguing species‐dependent differences and highlights the complexity and context dependence of venomous insect behavior.

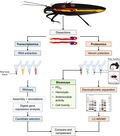

## 1 INTRODUCTION

The use of venom for predation and defense is common in the animal kingdom (Case well, Wüster, Vonk, Harrison, & Fry, 2013). Venoms produced by snakes, spiders, scorpions, sea anemones, and cone snails have been investigated in detail because they are toxic toward vertebrates and thus medically relevant (King, 2015). Among insects, research has focused mainly on Hymenoptera because their venoms are often allergenic and pose a risk of fatal anaphylaxis in humans (Bonifazi et al., 2005; Müller, 2010). In contrast, the suborder Heteroptera has been largely overlooked, although some species can inflict severe defensive bites on humans when disturbed (Haddad, Schwartz, Schwartz, & Carvalho, 2010; dos Santos, de Souza, Zanette, da Silva, & Strussmann, 2019). The Heteroptera are a diverse group of phytophagous, zoophagous, and hematophagous species that have adapted to exploit many terrestrial, aquatic, and semiaquatic habitats (Henry, 2009; Schuh & Weirauch, 2020). It is likely that the divergence of Heteroptera from more basal Hemiptera was accompanied by a shift to zoophagy, although some groups later shifted back to phytophagy (Johnson et al., 2018). The composition and evolution of Heteropteran venoms was recently reviewed in detail by Walker, Weirauch, Fry, and King (2016).

All heteropteran species feature piercing/sucking mouthparts, allowing them to inject salivary secretions into their food and suck up the liquid components (Cohen, 1998; Panfilio & Angelini, 2018). The saliva facilitates the extra‐oral digestion of solid tissues and therefore improves access to nutrients (Cohen, 1998). Proteases have an essential role in nonrefluxing extra‐oral digestion (Cohen, 1993, 1998) and are abundant in the saliva of numerous heteropteran species (Boyd, Cohen, & Alverson, 2002; Swart, Deaton, & Felgenhauer, 2006; Walker, Hernández‐Vargas, Corzo, Fry, & King, 2018). For example, in the salivary secretions of the Australian assassin bug *Pristhesancus plagipennis*, 69 of 127 enzymes are associated with proteolysis, whereas only three have putative functions in lipid catabolism, one in nucleic acid catabolism, and 10 proteins are associated with cytolysis (Walker et al., 2017).

In zoophagous Heteroptera, the salivary glands (also called venom glands) not only secrete enzymes for the digestion of animal tissue (Cohen, 1995, 1998) but also proteins and peptides that facilitate the capture of prey (Edwards, 1961; Walker et al., 2017, 2019). The rapid paralysis of insects attacked by predatory assassin bugs such as *Rhinocoris carmelita* Stål and *Platymeris rhadamanthus* Gerstaecker was initially attributed to the disruption of cell membranes by digestive enzymes rather than the action of neurotoxins (Edwards, 1961). However, the subsequent analysis of assassin bug salivary peptides revealed similarities to the neurotoxic peptide ω‐conotoxin from cone snails (Corzo, Adachi‐Akahane, Nagao, Kusui, & Nakajima, 2001). Further characterization of Ptu1, a peptide from the reduviid species *Peirates turpis* Walker, revealed the presence of an inhibitor cystine knot (ICK) motif that causes the reversible inhibition of Cav2.2 voltage‐gated calcium channels (Bernard, Corzo, Mosbah, Nakajima, & Darbon, 2001), thus refuting the hypothesis put forward by Edwards (1961). Recently, several Ptu1‐like peptides were identified in the salivary secretions of *P. plagipennis* Walker and *P. rhadamanthus* (Walker et al., 2017, 2019). The venoms from both reduviid species induced rapid paralysis when injected into insects (Walker, Mayhew, et al., 2018; Walker et al., 2019).

Many predatory bugs use venom not only to attack and digest prey but also defensively when they are disturbed. Backswimmers (Notonectidae), also called water bees, occasionally inflict painful bites on humans during swimming (Diaz, 2016). Bites inflicted by assassin bugs and belostomatids are extremely painful, and can trigger various symptoms including edema (Haddad et al., 2010; Hartwig, 1977; dos Santos et al., 2019), paresthesia and pruritus (dos Santos et al., 2019), and pseudoparalysis (Haddad et al., 2010). Such defensive bites mainly target vertebrates and probably fulfill different functions compared to bites administered when killing and digesting invertebrate prey. Predatory bugs may therefore produce distinct venom components that are specifically active against invertebrates and vertebrates, or even different types of venom for each purpose (Haridass & Ananthakrishnan, 1981; Walker et al., 2017).

Hemipteran salivary glands comprise two accessory glands and a pair of principal glands that typically feature an anterior main gland (AMG) and a larger posterior main gland (PMG) as distinct lobes (Baptist, 1941). The effects of reduviid gland homogenates on arthropods were shown to depend on the source, with AMG extracts causing paralysis and PMG extracts failing to induce paralysis but leading to death after a few hours, suggesting that AMG venom is used for prey immobilization whereas the PMG secretes digestive enzymes. In contrast, accessory gland homogenates did not show any effects when injected into prey (Haridass & Ananthakrishnan, 1981). The analysis of venom collected from *P. plagipennis* by electrostimulation revealed the presence of both neurotoxic peptides and digestive enzymes in the secretions (Walker et al., 2017), but more detailed analysis showed that the AMG and PMG secretions differ substantially, and are used for defense and prey killing/digestion, respectively (Walker, Mayhew, et al., 2018). The deployment of functionally distinct venoms has also been reported in scorpions (Inceoglu et al., 2003) and cone snails (Dutertre et al., 2014).

The defensive venom of *P. plagipennis* mainly consists of hemolysin‐like proteins, protease inhibitors, and several novel and uncharacterized proteins (Walker, Mayhew, et al., 2018). However, the mode of action of these secretions is largely unknown and requires further investigation. Furthermore, it remains unclear how the differential use of AMG and PMG venom is regulated in the insects and which ecological stimuli trigger the release of the specific venom types. In contrast to *P. plagipennis*, a recent study of the red spot assassin bug *P. rhadamanthus* showed that it uses PMG venom for both prey killing and defense (Walker et al., 2019). However, the authors only analyzed the defense spray and not the venom that is injected defensively by *P. rhadamanthus*. Thus, it is unclear whether the defensive use of AMG venom is unique to *P. plagipennis* or if there are more species with this remarkable adaptation. Furthermore, the hypothesis that *P. plagipennis* AMG venom is specialized for defense remains to be tested.

**Figure 1:**
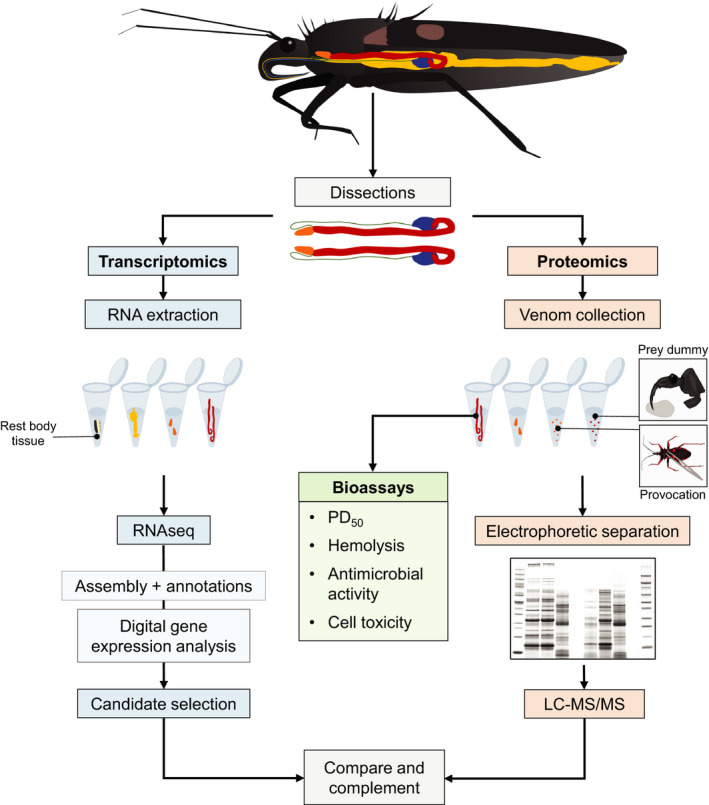
Schematic workflow of an integrated transcriptomic, proteomic and assay‐based approach to identify the venom‐specific proteins and venom activity of *P. biguttatus* and *P. horrida*

To gain insight into the context‐dependent deployment of venom by reduviid bugs and corresponding differences in venom composition, we conducted an integrated transcriptomics and proteomics analysis (Figure 1) to identify and compare the venom components of the reduvine species *Platymeris biguttatus* L. and *Psytalla horrida* Stål. Both species are native to western Africa, with overlapping habitats and a similar prey range (Chłond, Bugaj‐Nawrocka, & Junkiert, 2015; Gordon, 2017; Guilbert & Chłond, 2009). Ecological niche modeling revealed that *P. biguttatus* prefers tropical savanna as well as open areas with tree vegetation and shares potentially suitable niches with *P. rhadamanthus* (Chłond et al., 2015). We compared AMG and PMG extracts from the two species and confirmed that the PMG is the glandular origin of prey‐killing venom in both species. However, the analysis of venoms secreted in response to different stress stimuli revealed that *P. horrida* secretes both PMG and AMG venoms defensively in a context‐dependent manner, whereas *P. biguttatus* defensive secretions originate mostly from the PMG. We carried out a comprehensive analysis of venom components from both species and also conducted in vitro and in vivo bioactivity assays to investigate their effects. Our results contribute to a better understanding of venom deployment and function and provide a basis for further studies that will unravel the ecology of predatory Heteroptera and identify venom components with potential applications in medicine and agricultural pest control.

## 2 METHODS

### 2.1 Insects

*Psytalla horrida* and *P. biguttatus* specimens were obtained from an insectarium breeding source (Jörg Bernhardt, personal communication) and kept at room temperature in terraria laid out with sand, coconut fibers, and pieces of bark as hiding places. The bugs were fed once per week with *Acheta domesticus* L. or *Galleria mellonella* L. larvae, both of which were obtained from Tropic Shop (Nordhorn, Germany). Venom injection assays were carried out using *G. mellonella* obtained from BioSystems Technology (Exeter, UK).

### 2.2 Venom collection

Venom likely to have a defensive function was obtained by exposing the insects to different forms of stress, including mild harassment, cold stress, and the more intense harassment of restrained bugs. For mild harassment, the insects were separated in plastic boxes and prodded with forceps, but were not restrained and were allowed to escape. In most cases, individual bugs did not attack the forceps but a small droplet of saliva emerged at the proboscis tip, which could be collected using a pipette tip and transferred to a precooled 1.5‐ml Eppendorf tube. For cold stress, the bugs were exposed to −20°C for 3 min, which induced salivation. The droplet that emerged at the proboscis tip was also collected and transferred to a separate precooled tube. For strong harassment stress, a cold‐anaesthetized assassin bug was fixed on a foam cuboid and the proboscis was inserted into a pipette tip. When the insect was fully awake, it was tapped and gently squeezed with forceps, which induced salivation. The collected venom was transferred to a precooled tube.

In order to collect the saliva that *P. horrida* and *P. biguttatus* inject into their prey, an artificial prey dummy was prepared by enclosing a droplet of phosphate‐buffered saline (PBS), typically 20–60 µl, in a piece of stretched Parafilm. The prey dummy was held in front of the bugs to simulate moving prey insects and induce hunting behavior. When the bug attacked the artificial prey, it was allowed to inject saliva for 1.5 min before removing the dummy. The PBS‐venom mixture was recovered from the dummy and transferred to a precooled tube.

In addition to the noninvasive collection of saliva, venom was also extracted directly from the venom glands of fifth‐instar or adult assassin bugs that were separated and anaesthetized at −20°C for 5 min before dissection in PBS. The posterior and anterior lobes were separated and immediately placed in precooled tubes containing 10–40 µl PBS on ice. The samples were briefly vortexed and centrifuged (4,000 *g* for 3.5 min at 4°C), and the supernatant was transferred to a fresh tube. The venom of several individuals was pooled and stored at −20°C for analysis. The total protein concentration in the venom samples was measured using an N60 nanophotometer (Implen).

### 2.3 Proteomic analysis

The venom proteins were separated by sodium dodecylsulfate polyacrylamide gel electrophoresis (SDS‐PAGE) on 4%–12% Criterion XT gradient gels (BioRad) with XT MES running buffer at 125 V for 1.5 hr. Prestained and unstained high‐mass‐precision protein markers were used to determine the molecular weight (kDa) of the venom proteins. Gels were stained with a 1:1 mixture of Coomassie Brilliant Blue R‐250 and colloidal Coomassie Brilliant Blue G‐250 (Thermo Fisher Scientific) for 1.5 hr. Excess dye was removed by washing in Millipore water overnight, and the stained gel was then scanned and analyzed.

For LC‐MS/MS analysis, protein bands from each gel lane were excised as 29 molecular weight fractions for tryptic digestion (Shevchenko, Tomas, Havli, Olsen, & Mann, 2006). Further details of LC‐MS sample processing, data acquisition and data processing, such as search parameters specifying mass measurement accuracy, minimum number of product ion matches per peptide, minimum number of product ion matches per protein, minimum number of peptide matches, and maximum number of missed tryptic cleavage sites can be found in Methods S1, Section 1.

### 2.4 Venom gland collection and RNA isolation

The anterior and posterior lobes of the venom gland complex from fifth‐instar or adult assassin bugs, dissected as described above for venom collection, were placed in separate ceramic bead tubes containing 500 µl of TRI Reagent (Sigma‐Aldrich). The alimentary canal was carefully removed and also transferred into 500 µl of TRI Reagent. Finally, the fat body, muscle tissue, and integument were combined as the “remaining body tissue” and placed in a separate tube with TRI Reagent. The tissues of two individuals were pooled and homogenized using a TissueLyser LT (Qiagen). Total RNA was extracted using the Direct‐zol RNA Miniprep Kit according to the manufacturer's instructions (Zymo Research). The quantity of RNA was measured using a N60 nanophotometer, and its integrity was confirmed using an Agilent 2100 Bioanalyzer and RNA Nanochip (Agilent Technologies).

### 2.5 RNA‐SEQ and de novo transcriptome assembly

For both species, the AMG, PMG, gut, and remaining body tissue transcriptomes were sequenced by the Max‐Planck Genome Center Cologne (http://mpgc.mpipz.mpg.de/home/) using an Illumina HiSeq3000 Genome Analyzer platform. Poly‐A mRNA was isolated from 1 µg of total RNA using oligo‐dT attached to magnetic beads and fragmented to an average of 250 bp before sequencing libraries were generated using the TruSeq RNA Library Preparation Kit v2 (Illumina). Paired‐end (2 × 150 bp) read technology was used for sequencing, resulting in the following numbers of reads: *P. biguttatus* AMG = 50 million, PMG = 58 million, gut = 45 million, and remaining body tissue = 46 million; *P. horrida* AMG = 65 million, PMG = 68 million, gut = 51 million, and remaining body tissue = 60 million. All reads generated by the sequencing provider were processed using an in‐house assembly and annotation pipeline. The presumed optimal consensus transcriptome for each species was then selected, as previously described (Vogel, Badapanda, Knorr, & Vilcinskas, 2014). Details of the transcriptome assemblies, transcript annotation, and RNA‐Seq mapping can be found in Methods S1, Section 2.

### 2.6 Venom activity bioassays

To investigate the effects of *P. horrida* and *P. biguttatus* venom on prey insects, *G. mellonella* larvae were injected with AMG or PMG venom in preliminary tests, which showed that only PMG venom had any effect. Only PMG venom was therefore used in further experiments. We injected 5 µl of various concentrated PMG venom samples into the first proleg of *G. mellonella* larvae using a DMP microsyringe pump (World Precision Instruments). For *P. horrida* venom, we tested protein concentrations of 0.8, 1.2, 1.6, and 2.0 µg/µl, and for *P. biguttatus* venom, we tested protein concentrations of 0.6, 1.0, 1.4, and 2.0 µg/µl. We injected 5 µl of PBS as a negative control. Treated insects were observed 1 min, 1 hr, and 24 hr postinjection, and their behavior was recorded. We differentiated between normal larval behavior, partial paralysis, complete paralysis, and death. PD_50_ values were calculated with a logistic model in R v3.6.0 using the HelpersMG package and were based on the observation of larvae that were completely paralyzed or only able to move their legs and/or mandibles.

Hemolytic activity was tested on blood agar plates. On each plate, seven holes were punched out using a sterile 5‐mL pipette tip and the wells were filled with 2 µl of various concentrated venom extracts (PMG venom = 100, 20 or 1 µg/µl; AMG: venom = 20 or 1 µg/µl), 2 µl PBS as a negative control, or 2 µl 1% Triton‐X‐100 in water as a positive control. Hemolysis was tested on human blood agar, horse blood agar, and sheep blood agar plates. For each blood type and reduviid species, we prepared triplicates. The plates were incubated at 37°C for 24 hr and then photographed and inspected for hemolytic zones.

Bacterial growth inhibition was tested using a bacterial inhibition zone assay with *Escherichia coli*. Overnight cultures in lysogeny broth (LB) medium were prepared by inoculating 5 ml of fresh medium with one colony of *E. coli* and incubating overnight at 37°C. We transferred 100 µl of the overnight culture into 100 ml of warm LB agar, and plates were poured using 10 ml per Petri dish. On each plate, seven holes were punched out using a sterile 5 ml pipette tip and the wells were filled with 2.5 µl of various concentrated venom extracts (PMG venom = 80, 16 or 0.8 µg/µl; AMG venom = 16 or 0.8 µg/µl), 2.5 µl sterile PBS as a negative control, or 2.5 µl gentamycin (50 mg/ml) as a positive control. For each reduviid species, we prepared triplicates. The plates were incubated at 37°C for 24 hr and then photographed and inspected for bacterial growth inhibition zones.

Potential cytotoxic effects were tested on *Spodoptera frugiperda Sf*9 cells. The cells were cultured in Sf‐900 II SFM medium (Gibco) and seeded in Petri dishes with a diameter of 6 cm. After 24 hr, the culture medium was replaced with fresh medium containing 50 µl diluted venom (5 µg/µl) or 50 µl sterile PBS (negative control) and the plates were incubated at 27°C for 24 hr. The cells were then examined by phase‐contrast microscopy for cytotoxic effects.

## 3 RESULTS

### 3.1 Differential effects of AMG and PMG venom on *G. mellonella* larvae

**Figure 2:**
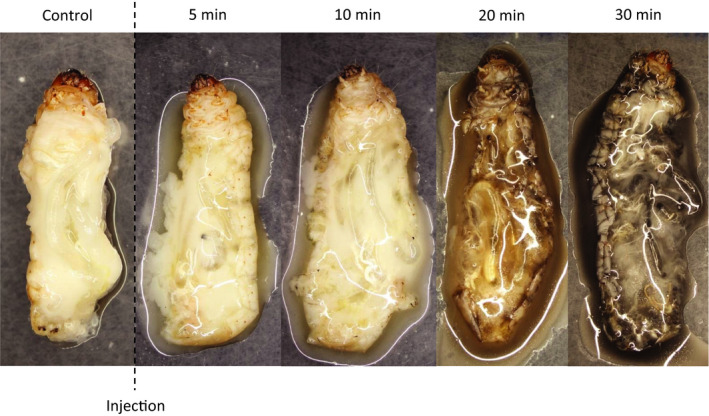
Digestive effects of *P. biguttatus* PMG venom on *G. mellonella* larvae at different time points after venom injection

Venom was extracted from the separated anterior and posterior lobes of *P. biguttatus* and *P. horrida* salivary glands by low‐speed centrifugation. Injections of AMG venom had no effect on *G. mellonella* larvae (data not shown) so we focused on the effects of PMG venom, which caused rapid paralysis and death. With an estimated PD_50_ of 6.2 µg per larva (23.4 µg/g) after 1 hr, the *P. biguttatus* PMG secretions were more potent than those from *P. horrida*, with an estimated PD_50_ of 9.8 µg per larva (37.0 µg/g). The digestive effects of PMG venom were confirmed by allowing *P. biguttatus* to inject venom into *G. mellonella* larvae, removing the prey after 1.5 min, and examining the condition of inner structures at different time points. The larvae began to melanize and liquefy after 20 min, and most structures were almost fully digested after 30 min (Figure 2). These effects clearly indicated the presence of strong paralytic components and digestive enzymes in the PMG secretions.

**Figure 3:**
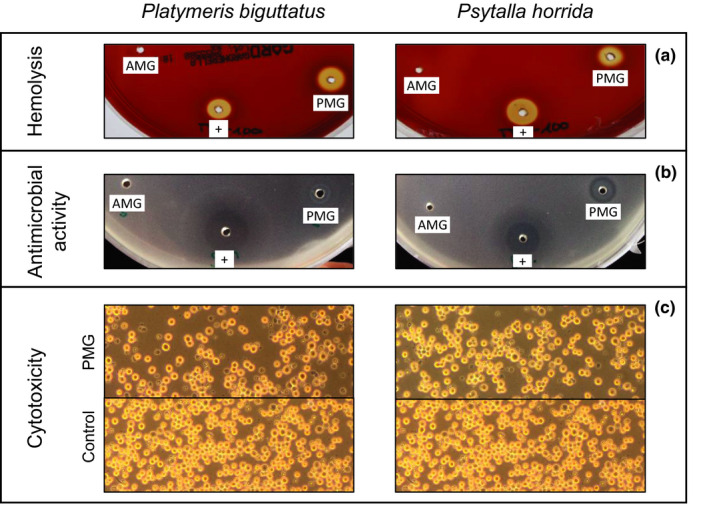
Hemolytic (a), antimicrobial (b), and cytotoxic (c) effects of PMG venom extracted from *P. biguttatus* and *P. horrida*. (a) AMG = 20 µg/µl anterior main gland venom; PMG = 100 µg/µl posterior main gland venom; + = 1% Triton X‐100. PMG venom generated large hemolytic zones in blood agar plates, indicating the presence of proteins with strong hemolytic activity. (b) AMG/PMG as above; + = 0.5 µg/µl gentamycin. PMG venom from either species caused the significant inhibition of bacterial growth in an *E. coli* inhibition zone assay. (c) Diluted PMG venom displayed cytotoxic activity against Sf9 cells, reducing the cell density and causing extensive cell death

Hemolysis, cytotoxicity, and antibacterial assays were carried out to characterize the activity of the AMG and PMG venoms in more detail. We found that the AMG venom (from both species) had no effect in any of the assays (data not shown). Hemolysis was tested on blood agar plates containing human, horse, or sheep erythrocytes. We found that 200 µg of *P. biguttatus* PMG venom generated large hemolytic zones on human and horse blood and also showed weak hemolytic activity against sheep blood. The same amount of PMG venom from *P. horrida* also showed strong hemolytic activity against human blood, but weaker effects against horse blood, and no activity against sheep blood (Figure 3a). These results indicated the presence of proteins with strong hemolytic activity in PMG venom. The application of 200 µg PMG venom from either species caused the significant inhibition of bacterial growth in an *E. coli* inhibition zone assay (Figure 3b). Finally, we tested for cytotoxic activity by exposing *Sf*9 cells to diluted venom extracts. We found that a concentration of 0.005 µg/µl of PMG venom from either species was cytotoxic, reducing the cell density and causing extensive cell death (Figure 3c). Taken together, these results indicate that PMG (but not AMG) secretions from *P. horrida* and *P. biguttatus* display neurotoxic, digestive, hemolytic, antibacterial, and cytotoxic effects, indicating different functional adaptations of the two types of venom.

### 3.2 Spatial separation of venoms for defense and prey killing

In *P. plagipennis*, which produces defensive venom in the AMG and prey‐killing venom in the PMG (Walker, Mayhew, et al., 2018), defensive venom can be collected by harassment and prey‐killing venom by electrostimulation (Walker et al., 2017; Walker, Rosenthal, Undheim, & King, 2018). However, electrostimulation is an artificial situation, and the resulting venom may differ from that injected into prey. We therefore established a new method for the collection of saliva using a Parafilm prey dummy filled with PBS, allowing the isolation and further analysis of prey‐killing venom. Defensive venom was collected by exposing insects to different forms of stress: mild harassment, cold stress, and the more intense harassment of restrained bugs.

**Figure 4:**
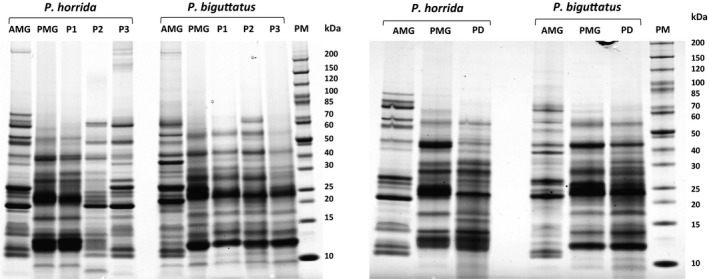
SDS‐PAGE analysis of venom extracts from homogenized glandular tissue and venom collected without dissection from *P. horrida* and *P. biguttatus*. AMG = anterior main gland extract; PMG = posterior main gland extract; P1 = provocation venom (mild harassment); P2 = provocation venom (cold stress); P3 = provocation venom (strong harassment); I1 = injection venom (prey dummy) after 1.5 min; and PM = protein marker

In our initial experiments, we compared the protein content of AMG and PMG extracts in each species to the content of the venoms collected from the prey dummies and stress‐induced secretions (Figure 4). In both species, the protein bands in the prey‐killing venom were similar to those in the PMG extracts, indicating that the posterior lobe is the glandular origin of the venom used to paralyze and digest prey. In *P. biguttatus*, the defensive venom samples were also similar to the PMG extract, whereas the composition of *P. horrida* defensive venom was context‐dependent. Venom obtained by mild harassment was similar to the PMG extract, whereas venom collected during cold stress or strong harassment clearly resembled the AMG extract. The results confirmed that *P. horrida* and *P. biguttatus* produce different types of venom in the AMG and PMG, which to a certain extent fulfill the roles of defensive and prey‐killing venoms, respectively.

**Figure 5:**
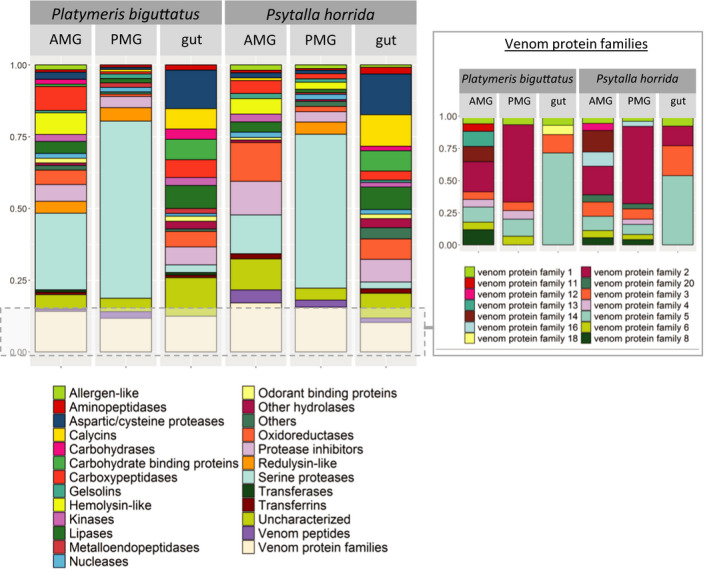
Protein composition of the AMG, PMG, and gut secretions of *P. biguttatus* and *P. horrida*. Color‐coded blocks show the number of contigs identified in transcriptome datasets encoding specific classes of functional proteins. The venom protein families are shown separately in the inset box

**Figure 6:**
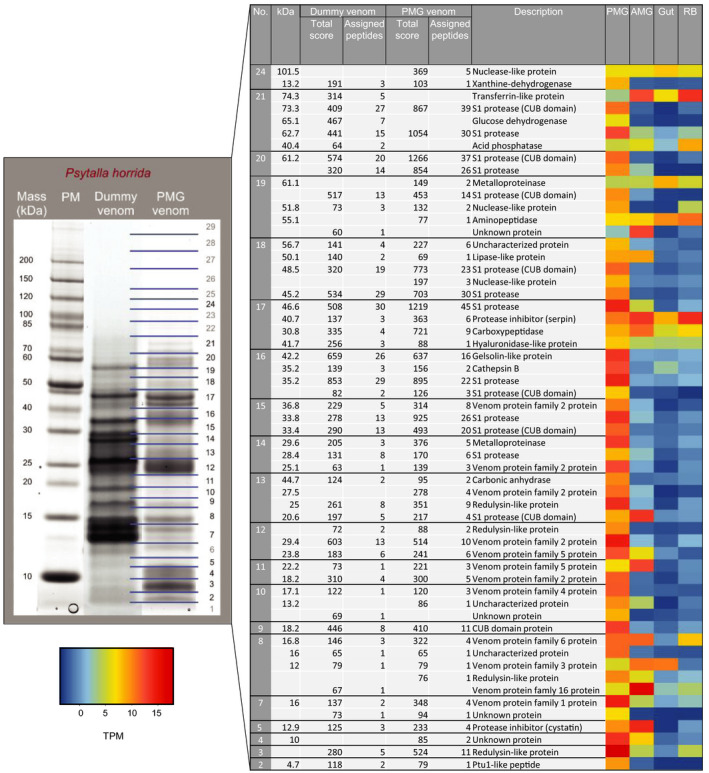
Proteins of the *P. horrida* PMG and prey dummy venom identified by LC‐MS/MS. The Coomassie‐stained protein gel on the left yielded the PMG venom proteins shown on the right, including the predicted protein masses (kDa), the total score, number of assigned peptides and descriptions. The excised bands are indicated with numbers and lines on the right side of the protein gel. For the proteins identified by LC‐MS/MS, gene expression levels (log2 TPM) in the PMG, AMG, gut, and remaining body tissues are shown in the heat map. PM = protein marker. See Table S1 for the identity of matching predicted proteins in the *P. horrida* transcriptome

**Figure 7:**
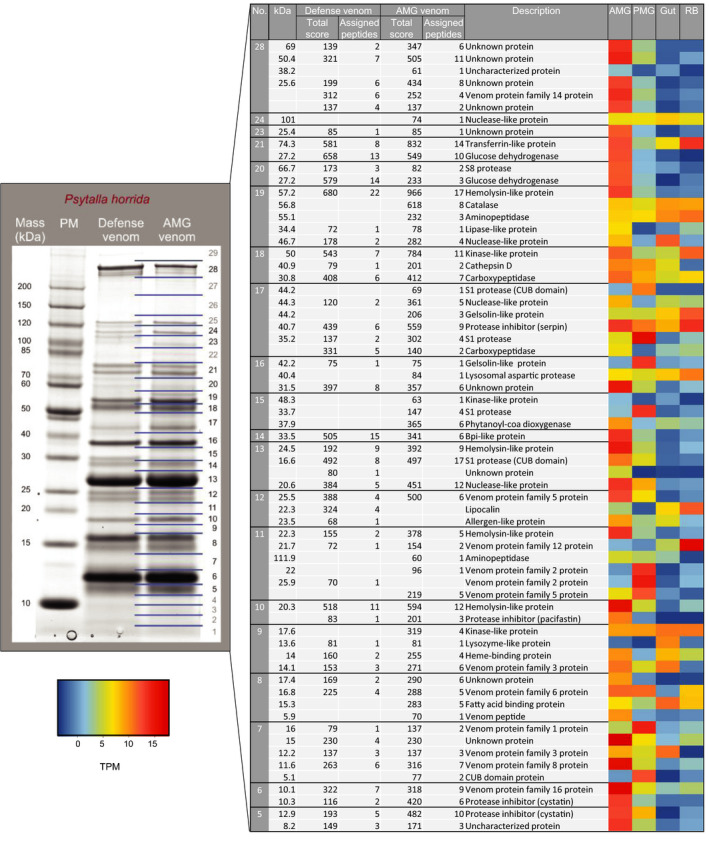
Proteins of the *P. horrida* AMG and defense venom (mild harassment) identified by LC‐MS/MS. The Coomassie‐stained protein gel on the left yielded the AMG venom proteins shown on the right, including the predicted protein masses (kDa), the total score, number of assigned peptides and descriptions. The excised bands are indicated with numbers and lines on the right side of the protein gel. For the proteins identified by LC‐MS/MS, gene expression levels (log2 TPM) in AMG, PMG, gut, and remaining body tissues are shown in the heat map. PM = protein marker. See Table S1 for the identity of matching predicted proteins in the *P. horrida* transcriptome

Protein bands from the AMG and PMG extracts, the strong harassment venom, and the prey‐killing venom were excised from the gel, digested with trypsin, and analyzed by LC‐MS/MS. The predicted peptide sequences were searched against translated ORFs from the *P. horrida* and *P. biguttatus* transcriptome datasets described below (Figure 5). In both species, the prey‐killing venom proteome was highly similar to the PMG proteome (Figure 6, Figure S1). In *P. horrida*, the defensive (strong harassment) venom proteome was more similar to the AMG proteome (Figure 7), whereas the defensive venom proteome of *P. biguttatus* mainly contained proteins from the PMG, along with some AMG proteins (Figure S2). Proteomic analysis therefore supported the theory that PMG secretions in both *P. horrida* and *P. biguttatus* are used mainly for prey killing, whereas AMG secretions in *P. horrida* serve as defensive venom in response to strong harassment.

### 3.3 Gene expression and protein composition of PMG and AMG venom glands

Further evidence for the protein composition of PMG and AMG venom was obtained by next‐generation sequencing (RNA‐Seq), allowing the identification and quantitation of venom‐associated transcripts. RNA was isolated from the AMG, PMG, gut, and remaining body tissue of both species for Illumina sequencing, which yielded 45–68 million reads per sample. The de novo reference transcriptome assembly for *P. biguttatus* contained 47,377 contigs, with an N50 contig size of 1,579 bp and a maximum contig length of 20,166 bp, whereas the equivalent assembly for *P. horrida* contained 37,424 contigs, with an N50 contig size of 1,623 bp and a maximum contig length of 20,054 bp. BUSCO analysis revealed 91.9% and 92.4% complete gene coverage in addition to 2.3% and 1.8% missing genes for the *P. biguttatus* and *P. horrida* transcriptome assemblies, respectively. The contigs were screened against the nonredundant NCBI protein database, and functional annotations were added. For digital gene expression analysis, the Illumina reads were remapped onto the assemblies to calculate expression values. Although comparisons of RPKM and TPM levels revealed no major differences, we used the log2 TPM value for between‐tissue comparisons. Potential venom‐associated contigs were selected based on their BLAST hits, annotations, and expression levels. Furthermore, the candidate proteins were checked for signal peptides and matched against the proteome data (see above). We ultimately selected 128 (PMG) and 120 (AMG) venom protein candidates for *P. biguttatus* as well as 166 (PMG) and 111 (AMG) for *P. horrida*. The candidates were classified according to their domains, predicted molecular functions, and family memberships.

The venom gland transcriptomes of *P. horrida* and *P. biguttatus* are compared in Figure 5. The comparative profiles of the AMG, PMG, and gut transcriptomes revealed major tissue‐specific but only minor species‐dependent differences. Most of the PMG venom transcripts in both species could be assigned to S1 family proteases (with many containing a CUB domain) or the different venom protein families identified in *P. plagipennis* (particularly venom protein families 1, 2, 3, 4, 5, and 6). The PMG transcriptome of *P. horrida* also contained matches to venom protein families 8 and 20. Furthermore, we found six (*P. biguttatus*) and seven (*P. horrida*) redulysin‐like sequences in the PMG transcriptome among several other groups including gelsolins, protease inhibitors, and Ptu1‐like peptides (Figure 5). The contig with the highest PMG‐specific expression in both species encoded a redulysin‐like protein, followed by S1 proteases and proteins from venom protein families 1 and 2 (Figure 6, Figure S1). Other abundant transcripts in the PMG transcriptome encoded a gelsolin‐like protein, a metalloproteinase, and other venom protein family members. We identified three (*P. biguttatus*) and four (*P. horrida*) ICK family peptides in the transcriptome datasets, but only one was also detected in the PMG proteome. This Ptu1‐like peptide was homologous to a peptide in *P. plagipennis* and showed strong PMG‐specific expression in both *P. biguttatus* and *P. horrida*. The main components and composition of the PMG venom therefore appeared to be similar in both species, although more complex in the case of *P. horrida*.

The AMG venom showed more species‐dependent differences in composition. S1 proteases and venom protein family members were predominant in both species, but the *P. biguttatus* AMG transcriptome featured more than double the number of S1 protease sequences compared to *P. horrida*. Venom protein families 1, 2, 3, 5, 6, 8, and 14 were represented in both species, whereas venom protein families 4, 11, and 13 were specific to *P. biguttatus* and venom protein families 12, 16, and 20 were specific to *P. horrida*. We also identified transcripts representing several hemolysins, protease inhibitors (including cystatins, serpins, and pacifastins), and an odorant‐binding protein in both species (Figure 5). In *P. horrida*, the contig with the highest AMG‐specific expression encoded a member of venom protein family 16, followed by venom protein family 8, a hemolysin, and two cystatin and pacifastin protease inhibitors (Figure 7). In *P. biguttatus*, the contig with the highest AMG‐specific expression encoded a hemolysin, followed by an uncharacterized protein, two protease inhibitors (cystatin and pacifastin), and an S1 protease with a CUB domain (Figure S2). In both species, the AMG appeared to secrete a more complex protein mixture than the PMG.

**Figure 8:**
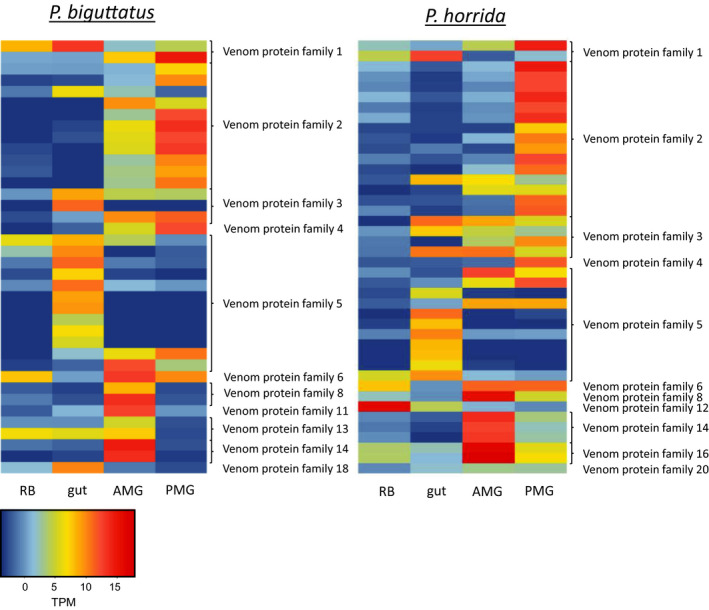
Gene expression levels (log2 TPM) of proteins from different venom protein families in the rest of body tissue (RB), gut, AMG, and PMG for *P. biguttatus* and *P. horrida*

Looking more closely at the venom protein families, we observed some groups that were present in the venom gland and gut transcriptomes (Figure 5). The majority of these tissue‐wide venom protein families were members of venom protein families 3 and 5, with transcripts in the AMG and PMG, and several that were solely expressed in the gut (Figure 8). Members of venom protein families 1, 2, and 18 were also found in the gut transcriptomes (Figure 8). The role of these venom protein family proteins is not clear, but it is likely that not all of them possess venom‐specific functions.

The venom glands of both *P. horrida* and *P. biguttatus* produced several uncharacterized proteins and unknown proteins with no hits in the protein database. Some of them were identified on the basis of specific domains or Interpro family memberships, and others via BLAST searches of the translated ORF containing the sequence predicted by LC‐MS/MS. However, most of these proteins could not be assigned to any known family or associated with any specific domain or motif. The *P. horrida* AMG in particular featured many unknown transcripts with strong tissue‐specific expression representing proteins that were abundant in the corresponding proteome (Figures 5 and 7). The functions of these abundant proteins should be investigated in future experiments. In both species, some transcripts were strongly expressed not only in the venom glands but also in the gut and remaining body tissues. These included transcripts encoding transferrins, serpins, kinases, carboxypeptidases, and aminopeptidases. The proteins were also present in the AMG and/or PMG proteomes, indicating they are genuine secreted proteins that might play an important role in the venom secretions. However, their expression in nonglandular tissues suggests they possess additional functions that are unrelated to the effects of AMG and PMG venom.

### 3.4 Differentiation of internal and extra‐oral digestion

Predatory bugs cannot take up solid food and must predigest their prey extra‐orally before sucking up the liquefied tissues for further digestion in the gut. The properties of extra‐oral digestion enzymes differ from those of enzymes in the gut in terms of pH preference and target substrates. Given that the PMG secretions of *P. horrida* and *P. biguttatus* are required for prey killing and digestion, we compared the PMG and gut transcriptomes of both species and found that the two tissues secrete different sets of proteins (Figure 5). Specifically, serine proteases were predominant in the PMG transcriptomes whereas cysteine and aspartic proteases were more common in the gut. Furthermore, the gut transcriptomes also contained sequences encoding several lipase‐like proteins, carbohydrate‐binding proteins, and calycins that were not represented in the PMG. Interestingly, members of venom protein family 5 were also prevalent in the gut transcriptomes, indicating a potential digestive function.

## 4 DISCUSSION

To determine the glandular origins and utilization of different types of venom in the two reduviid species *P. biguttatus* and *P. horrida*, we used three nonlethal collection methods that mimic natural stimuli. Electrostimulation is often used to collect venom from arthropods, and this approach has been successful in Hymenoptera (Mueller et al., 1981), Heteroptera (Walker, Hernández‐Vargas, et al., 2018; Walker, Mayhew, et al., 2018), centipedes (Jenner, von Reumont, Campbell, & Undheim, 2019; Malta et al., 2008), spiders (Barbaro, Cardoso, Eickstedt, & Mota, 1992; da Silveira et al., 2002), and scorpions (Carcamo‐Noriega, Possani, & Ortiz, 2019; Rowe & Rowe, 2008). The advantage of electrostimulation is that it yields large volumes of venom (Glenn, Straight, & Snyder, 1972; Rocha‐e‐Silva, Sutti, & Hyslop, 2009) that is usually free from tissue contamination (Mueller et al., 1981). However, it is an unnatural stimulus, and secretions obtained in this manner may not always match the composition and effects of natural venom, as reported for the ant *Myrmecia pilosula* Smith (Wiese et al., 2008). We therefore established a more realistic method to collect prey‐killing venom using a prey dummy to mimic a natural attack scenario, which in our hands allows the collection of high‐quality venom from many Heteroptera. Defensive venom can be collected by harassment (Walker, Mayhew, et al., 2018; Walker, Rosenthal, et al., 2018), and similarly, we applied mild and intense harassment as well as cold stress in order to provoke the secretion of defensive venom by *P. biguttatus* and *P. horrida*. Finally, venom can be obtained directly by the extraction of dissected venom glands, although this method is lethal (Drenth, 1974; Heep et al., 2019; Laurino et al., 2016; da Silveira et al., 2002; Walker, Hernández‐Vargas, et al., 2018; Walker, Mayhew, et al., 2018). In contrast to whole gland homogenates clarified by high‐speed centrifugation, we found that low‐speed centrifugation (Walker, Mayhew, et al., 2018) produced clean extracts without tissue contamination by avoiding pressure‐induced cytolysis. The comparison of secretions from *P. plagipennis* collected using various stimuli and direct extracts from dissected AMG and PMG tissues revealed differences that allowed the secretions obtained by mild harassment to be defined as defensive venom and suggested that the lethal neurotoxic venom obtained by electrostimulation is probably used for prey immobilization and digestion (Walker, Mayhew, et al., 2018). Furthermore, Walker et al. (2019) found that the mild harassment of *P. rhadamanthus* induced venom spitting/spraying, which was defined as defensive venom.

The venoms of zoophagous bugs are used to paralyze and liquefy prey insects (Cohen, 1993; Edwards, 1961; Walker et al., 2017, 2019; Walker, Mayhew, et al., 2018), thus facilitating the ingestion of nutrients via the proboscis (Cohen, 1998). Recently, the PMG was identified as the glandular origin of prey‐killing venom in *P. plagipennis* and *P. rhadamanthus* (Walker, Mayhew, et al., 2018; Walker et al., 2019). Our integrated transcriptomics and proteomics approach confirmed that the PMG is also the source of prey‐killing venom in *P. biguttatus* and *P. horrida*. Protein bands of venom samples collected using the prey dummy were similar to the PMG extracts, and most of the prey‐killing venom proteins identified by LC‐MS/MS were also present in the PMG extracts (Figure 6, Figure S1). The corresponding transcripts were strongly expressed in a PMG‐specific manner confirming that the PMG is the source of venom used for prey immobilization and extra‐oral digestion in both species.

One of the key functions of heteropteran prey‐killing venom is the immobilization of prey. Heteropteran venoms were thought to lack neurotoxins (Azevedo et al., 2007; Edwards, 1961), but several putative neurotoxic peptides have been identified and isolated (Bernard et al., 2001; Corzo et al., 2001; Walker et al., 2017, 2019). These include Ptu1, an ICK family peptide isolated from the assassin bug *Peirates turpis* Walker, which can reversibly block Cav2.2 voltage‐gated calcium channels in a similar manner to the homologous ω‐conotoxins from cone snails (Corzo et al., 2001; Kasai, Aosaki, & Fukuda, 1987). The ICK motif features a cystine knot and an antiparallel, triple‐stranded β‐sheet (Lavergne, Alewood, Mobli, & King, 2015; Norton & Pallaghy, 1998; Pallaghy, Norton, Nielsen, & Craik, 1994). Such intra‐chain disulfide bonds often stabilize the tertiary structure of peptides in animal venoms (Lavergne et al., 2015). In our experiments, PMG extracts from *P. horrida* and *P. biguttatus* triggered rapid paralysis when injected into *G. mellonella* larvae, with low PD_50_ values of 9.8 µg (37.0 µg/g) and 6.2 µg (23.4 µg/g) total protein, respectively. This matches the paralytic effects of *P. plagipennis* and *P. rhadamanthus* PMG venom (Walker, Mayhew, et al., 2018; Walker et al., 2019). We identified three specific peptides homologous to *P. plagipennis* Ptu1‐like peptides in the PMG transcriptomes of *P. horrida* and *P. biguttatus*, and one peptide homologous to the ICK family peptide Ado1 from *Agriosphodrus dohrni* Signoret in the PMG transcriptome of *P. horrida*. Among these sequences, only one Ptu1‐like peptide was found in the PMG proteome and was present in both *P. horrida* and *P. biguttatus*. Given the similarity of these sequences to ICK peptides from other venoms, their strong gland‐specific expression, and the presence of signal peptides, we are confident that these other ICK peptides are secreted by *P. horrida* and/or *P. biguttatus*, but we did not detect them due to the limitations of the Bis‐Tris polyacrylamide gels used in our experiment. Uncharacterized proteins such as members of venom family 1 are also thought to possess neurotoxic activity in reduviid bugs (Walker et al., 2017). We identified tissue‐specific transcripts representing venom protein family 1 not only in the PMG and AMG, but also in the gut of both species, suggesting these sequences are unlikely to encode neurotoxic peptides and probably fulfill housekeeping or digestive functions. In contrast, an uncharacterized member of venom protein family 4 was specific to (and strongly expressed in) the PMG transcriptome and may therefore represent a novel neurotoxin.

Extra‐oral digestion is a common trait among zoophagous invertebrates, allowing even small predators to ingest large prey species (Cohen, 1995). Predatory Heteroptera achieve nonrefluxing extra‐oral digestion by injecting digestive enzymes from the salivary glands into their prey and sucking up the liquefied tissues (Cohen, 1998). Likewise, phytophagous species typically pre‐digest plant material before ingesting it (Mehrabadi, Bandani, & Dastranj, 2014; Zhu, Yao, & Luttrell, 2016). We observed the rapid digestion of *G. mellonella* larvae injected with *P. biguttatus* PMG venom, indicating the presence of efficient digestive enzymes. Endopeptidases, particularly serine proteinases, appear to play a key role during extra‐oral digestion by heteropteran insects and are abundant in the salivary secretions of phytophagous (Mehrabadi et al., 2014; Zhu et al., 2016), hematophagous (Amino, Tanaka, & Schenkman, 2001; Meiser et al., 2010), and zoophagous species (Bell, Down, Edwards, Gatehouse, & Gatehouse, 2005; Walker, Hernández‐Vargas, et al., 2018; Walker et al., 2017, 2019). We found that S1 proteases were predominant in *P. horrida* and *P. biguttatus* PMG venom, some with very high expression levels. We also identified one *P. horrida* dipeptidase, three *P. horrida* exopeptidases, and two *P. biguttatus* exopeptidases in the PMG transcriptomes and proteomes, but all were expressed nonspecifically. In contrast, mainly cysteine and aspartic endopeptidases (and several exopeptidases) were present in the gut transcriptomes. This indicates that extra‐oral digestion in the two reduviid species focuses on initial proteolysis by serine‐type endopeptidases so that further protein digestion by endopeptidases and exopeptidases can take place in the gut (Bell et al., 2005; Cohen, 1993). Extra‐oral digestion also breaks down lipids, especially cell membranes and storage lipids (Cohen, 1995). We identified one triacylglycerol lipase in the PMG of *P. horrida* and two in the PMG of *P. biguttatus*, but we found no phospholipases. Other strongly expressed lipase‐like proteins, including triacylglycerol lipases, carboxylesterases, and phospholipases, were found in the guts of both species. This indicates that extra‐oral digestion prioritizes the predigestion of storage lipids by triacylglycerol lipases, whereas most lipid catabolism, including the digestion of phospholipids, takes place in the gut. In contrast to other true bugs (Boyd et al., 2002; Swart et al., 2006; Zeng & Cohen, 2000), no carbohydrase‐like proteins were found in the PMG transcriptomes or proteomes of either species, but two glucosidases were present in the gut transcriptomes of both.

Heteropteran venoms need to fulfill several additional functions to overcome prey defenses (Ayyachamy, Sahayaraj, & Rivers, 2016; Sahayaraj & Muthukumar, 2011), improve the spread of venom (Edwards, 1961), and optimize nutrient availability (Cohen, 1995). Furthermore, the predator needs to protect itself from microbial colonization of the salivary gland complex and infections caused by the ingestion of pathogens. The saliva of *Rhynocoris* species triggers rapid hemolysis in its prey, thus suppressing initial defense mechanisms including hemocyte spreading and aggregation (Ayyachamy et al., 2016; Sahayaraj & Muthukumar, 2011). The venoms of *Rhynocoris marginatus* Fabricius and *Catamirus brevipennis* Servile suppress Gram‐positive and Gram‐negative bacteria, with greater efficacy against the latter (Sahayaraj, Borgio, Muthukumar, & Anandh, 2006). *P. rhadamanthus* venom increases calcium influx in mouse dorsal root ganglion cells, probably by forming pores in cell membranes (Walker et al., 2019). We tested the hemolytic, antimicrobial, and cytotoxic activity of *P. horrida* and *P. biguttatus* venoms and found that AMG venom displayed none of the abovementioned effects whereas PMG venom was able to lyse erythrocytes (with greatest efficacy against human cells), inhibit the growth of *E. coli*, and reduce the viability of cultured insect cells. Redulysin‐like proteins were abundant in the PMG venoms of both species, and have previously been identified in *P. plagipennis* and *P. rhadamanthus*, where they may act as pore‐forming proteins with a cytolytic motif (Walker et al., 2017, 2019). Redulysin‐like proteins are homologous to trialysin, a protein found in the saliva of the blood‐feeding reduviid *Triatoma infestans* Klug (Amino et al., 2002) that can lyse bacteria, protozoans, and mammalian cells (Amino et al., 2002; Martins et al., 2006). The PMG‐specific redulysins in *P. horrida* and *P. biguttatus* are probably responsible for the observed hemolytic, antimicrobial, and cytotoxic effects, but other components may also contribute. For example, we identified one strongly expressed PMG‐specific gelsolin‐like protein in both assassin bugs. Gelsolin facilitates membrane ruffling and cytoskeletal deconstruction by enhancing actin depolymerization (Harms et al., 2004; Sun, Yamamoto, Mejillano, & Yin, 1999), and gelsolin‐derived peptides are active against Gram‐negative and Gram‐positive bacteria (Bucki et al., 2004).

The use of separate venomous secretions for prey killing and defense is a rare trait only reported in scorpions (Inceoglu et al., 2003) and cone snails (Dutertre et al., 2014) until recently. The first insect shown to deploy separate venoms was the harpactorine bug *P. plagipennis*, which uses AMG venom for defense and PMG venom to subdue and digest prey (Walker, Mayhew, et al., 2018). However, this is not a common trait among reduviid species. *P. rhadamanthus* mostly uses PMG venom for prey killing and defense, leaving the role of AMG venom unclear (Walker et al., 2019). *P. biguttatus* appears to follow the same strategy, primarily secreting PMG venom (and small quantities of AMG venom) in response to different stress situations. In contrast, *P. horrida* appears to use its PMG and AMG venoms defensively in a context‐dependent manner. All three species belong to the subfamily Reduviinae, but the phylogenetic relationships between *P. rhadamanthus*, *P. biguttatus*, and *P. horrida* remain unclear because the subfamily is likely to be polyphyletic (Hwang & Weirauch, 2012; Weirauch & Munro, 2009). In our experiments, the PMG venom was secreted in response to mild harassment without restraint (allowing the bug to escape from serious confrontation), possibly representing the response to intraspecific conflict. The insects did not attack the forceps but secreted a droplet of venom that stuck to the proboscis tip. This may serve as warning behavior among conspecifics in order to avoid cost‐intensive fighting. In contrast to mild harassment, more intense stress (conceived as a predator attack) was countered by the secretion of AMG venom, which is inactive against insects but may be effective against vertebrates (Walker, Mayhew, et al., 2018). The use of AMG venom for defense against vertebrates in *P. plagipennis* (Walker, Mayhew, et al., 2018) matches our observations in *P. horrida* and indicates that it may be used to induce pain. We detected hemolysin‐like proteins that were strongly expressed specifically in the AMG of both *P. horrida* and *P. biguttatus*, but these are unlikely to possess hemolytic activity (despite their name) given the results of our functional assays. Such proteins may instead act on mast cells, thus triggering the release of pain‐inducing compounds (Schmidt, Blum, & Overal, 1983), although specific bioassays would be required to confirm this hypothesis. This is the mechanism used by melittin in bee venoms (Chen, Guan, Sun, & Fu, 2016; Tosteson & Tosteson, 1981) and α‐hemolysin produced by *Staphylococcus aureus* Rosenbach, which induces pain by binding to the receptor ADAM10 in nociceptor neurons and triggering calcium influx by pore formation (Chiu, 2018; Wilke & Wardenburg, 2010). It is unclear why *P. horrida* alone uses AMG venom defensively, whereas *P. biguttatus* relies mainly on PMG venom. One major difference between the AMG venoms of these species is the absence in *P. biguttatus* of venom protein family 16. A detailed functional characterization of this venom protein is necessary to determine its role in predator deterrence. Other AMG venom proteins that may fulfill important defensive functions of include cystatins, pacifastins, cysteine‐rich secreted proteins, and additional uncharacterized venom protein families. The specific function of the AMG in *P. biguttatus* remains unclear because our experiments did not find any evidence that AMG venom is used for defense. Future research should focus on the characterization of strongly expressed proteins and peptides that are restricted to the AMG or PMG, and should also look at the many uncharacterized venom protein families.

Our comprehensive analysis of venom composition, effects, and deployment by the two reduviid species *P. horrida* and *P. biguttatus* revealed intriguing species‐dependent differences in composition and context‐dependent use. Given that venom regeneration can take several days and uses energy reserves, venom deployment (and reservoir depletion) probably results in considerable disadvantages for the insects until the reserves are replenished (Morgenstern & King, 2013). The AMG is much smaller than the PMG and yields less venom, probably because the insects need less of it. *P. horrida* may deploy AMG secretions only as a last line of defense, such as when caught by a (vertebrate) predator or disturbed by larger animals, including humans. Such strategic use of defensive venom has also been observed in scorpions, which can adjust the amount of venom injected, the venom composition, and the frequency of stings according to threat levels (Nisani & Hayes, 2011). Although both reduviid species share the same habitat and prey range, and their AMG and PMG secrete similar cocktails of proteins, only *P. horrida* appears to distinguish between different threats and respond accordingly. It is unclear how the injection of specific venom types is regulated and whether the release of AMG venom by *P. biguttatus* can be triggered by stimuli other than those tested in our experiments. Our results highlight the complexity of assassin bug behavior and its context dependence. Furthermore, although the use of AMG secretions for defense is not restricted to the subfamily Harpactorinae, it is not a consistent trait among the Reduviidae and can clearly differ even between closely related species.

## CONFLICT OF INTEREST

None declared.

## AUTHOR CONTRIBUTIONS

**Maike L. Fischer:** Data curation (lead); Formal analysis (equal); Investigation (equal); Methodology (equal); Visualization (lead); Writing‐original draft (lead). **Natalie Wielsch:** Data curation (supporting); Methodology (supporting); Writing‐review & editing (supporting). **David G. Heckel:** Funding acquisition (supporting); Supervision (supporting); Writing‐review & editing (supporting). **Andreas Vilcinskas:** Conceptualization (supporting); Funding acquisition (equal); Supervision (supporting); Writing‐review & editing (equal). **Heiko Vogel:** Conceptualization (lead); Data curation (supporting); Formal analysis (equal); Funding acquisition (equal); Supervision (lead); Writing‐review & editing (lead).

## Supporting information

Supplementary Material

Table S1

## Data Availability

The short‐read data described herein have been deposited in the EBI Sequence Read Archive with accession numbers ERS4259175–ERS4259178 for *P. biguttatus* and ERS4259179–ERS4259182 for *P. horrida*. The complete study can also be accessed directly using the following URLs: http://www.ebi.ac.uk/ena/data/view/PRJEB36335 and http://www.ebi.ac.uk/ena/data/view/PRJEB36336. Supplemental Methods, Figures, and Table S1 with detailed proteomic data analysis information are deposited in the Open Access Data Repository EDMOND and can be directly accessed at the following https://dx.doi.org/10.17617/3.4b
