# Supplementary material for: Context‐dependent venom deployment and protein composition in two assassin bugs
Source: Ecol Evol. 2020 Aug 17;10(18):9932–47. doi: 10.1002/ece3.6652 (PMC7520181; doi:10.1002/ece3.6652)
Supplement: Supplementary file 1 — Supplementary Material [file ECE3-10-9932-s001.docx]

**Supplemental Information for:**

**Context-dependent venom deployment and protein composition in two assassin bugs**

Maike L. Fischer, Natalie Wielsch, David G. Heckel, Andreas Vilcinskas, Heiko Vogel

**Supplementary Methods**

**1. LC-MS/MS, proteomic data processing and protein identification**

The tryptic peptides were dissolved in 50 μL aqueous 1% formic acid and 1 to 5 µL of the peptide mixture was injected into a Waters M-class ultra-high performance liquid chromatography (UPLC) system coupled online to a Synapt G2-si mass spectrometer (Waters). Samples were pre-concentrated and desalted online using a UPLC M-Class Symmetry C18 trap column (100 Å, 180 µm x 20 mm, 5 µm particle size) and 0.1% aqueous formic acid at a flow rate of 15 µL min^-1^. The peptides were eluted onto an Acquity UPLC HSS T3 analytical column (100 Å, 75 µm X 200, 1.8 µm particle size) at a flow rate of 350 nL min^-1^ using a gradient of mobile phase A (0.1% aqueous formic acid) and B (acetonitrile plus 0.1% formic acid) increasing from 2-10% B over 5 min, 10–40% B over 40 min, 40–70% B over 7 min, 70-95% B over 3 min, isocratic at 95% B for 2 min, and a return to 1% B. The eluted peptides were injected into the mass spectrometer operating in V-mode and positive ESI mode, with a resolving power of at least 20,000 full width at half maximum height (FWHM). We injected 100 fmol μL^-1^ human Glu-fibrinopeptide B in 0.1% formic acid/acetonitrile (1:1 v/v) at a flow rate of 1 μL min^-1^ via the reference sprayer every 45 s to compensate for mass shifts in MS and MS/MS fragmentation mode.

Data were collected by data-dependent acquisition (DDA). The acquisition cycle for DDA analysis consisted of a survey scan covering the *m/z* range 400–1800 Da followed by MS/MS fragmentation of the 10 most intense precursor ions collected at 0.5-s intervals in the *m/z* range 50–2000. Dynamic exclusion was applied to minimize multiple fragmentations for the same precursor ions. MS data were collected using MassLynx v4.1 software (Waters).

DDA raw data were processed and screened against a sub-database containing common contaminants (human keratins and trypsin) using ProteinLynx Global Server (PLGS) v2.5.2 (Waters). The following parameters were applied: fixed precursor ion mass tolerance = 15 ppm for survey peptide, fragment ion mass tolerance = 0.02 Da, estimated calibration error = 0.002 Da, one missed cleavage, fixed carbamidomethylation of cysteine residues, and variable oxidation of methionine. Spectra that remained unmatched following the database search were interpreted *de novo* to yield peptide sequences for homology-based searching using MS BLAST (Shevchenko et al. 2001) installed on a local server. MS BLAST searches were performed against the Arthropoda database (download from NCBI on 12 February 2019) and *P. horrida* and *P. biguttatus* sub-databases obtained from *in silico* translation of the corresponding transcriptomes. The pkl files generated from raw data were searched in parallel against the NCBInr database (downloaded on 10 January 2019) combined with *P. horrida* and *P. biguttatus* sub‑databases using MASCOT v2.6.0 and the parameters described above.

**2.** **RNA-Seq and *de novo* transcriptome assembly**

Quality control measures, including the filtering of high-quality reads, the removal of reads containing primer/adaptor sequences, and the trimming of read lengths, were applied using CLC Genomics Workbench v11.1. For transcriptome assembly, RNA-Seq data from all four tissue samples were combined for each species and *de novo* transcriptome assemblies were prepared using CLC Genomics Workbench v11.1 with standard settings and two additional CLC-based assemblies with different parameters. The presumed optimal consensus transcriptome for each species was then selected, as previously described (Vogel et al., 2014). The transcriptomes were annotated using BLAST, Gene Ontology and InterProScan in OmicsBox (<https://www.biobam.com/omicsbox>) as described by Götz et al. (2008). For BLASTx searches against the non-redundant NCBI protein database (NR database), up to 20 best NR hits per transcript were retained, with an E-value cutoff of ≤10^-3^ and a minimum match length of 15 amino acids. To assess transcriptome completeness, we performed a Benchmarking Universal Single-Copy Orthologs (BUSCO) analysis (http://busco.ezlab.org) by comparing our assembled transcriptomes against a set of highly-conserved single-copy orthologs. This was accomplished using the BUSCO v3 pipeline (Waterhouse et al., 2017), comparing the predicted proteins of the *P. biguttatus* and *P. horrida* transcriptomes to the predefined set of 1658 Insecta single-copy orthologs from the OrthoDB v9.1 database. Digital gene expression analysis was carried out using CLC Genomics Workbench v11.1 to generate BAM files, and then counting the sequences to estimate expression levels, using previously described parameters for read mapping and normalization (Pöppel et al., 2015). Gene expression levels were estimated by normalizing mapped read values as implemented in CLC Genomics Workbench v11.1 and ArrayStar, calculating the reads per kilobase per million mapped reads (RPKM) and transcripts per million (TPM) values. For comparisons of expression levels between tissues, we used the log2 transformed TPM value.

Potential venom or gut transcripts were screened for the presence of a signal peptide using SignalP 4.1 and classified based on the annotations. The extracted tissue-specific gene sets were then compared and complemented with the proteins that were identified by LC-MS/MS analysis.

**Supplementary References:**

Götz S., Garcia-Gomez JM., Terol J., Williams TD., Nagaraj SH., Nueda MJ., Robles M., Talon M., Dopazo J. and Conesa A. (2008). High-throughput functional annotation and data mining with the Blast2GO suite. Nucleic acids research, 36(10), 3420-35.

Pöppel A-K, Vogel H, Wiesner J, Vilcinskas A. 2015. Antimicrobial peptides expressed in medicinal maggots of the blow fly Lucilia sericata show combinatorial activity against bacteria. Antimicrob Agents Chemother 59:2508 –2514. doi:10.1128/AAC.05180-14.

Shevchenko A, Sunyaev S, Loboda A, Bork P, Ens W, Standing KG. 2001. Charting the proteomes of organisms with unsequenced genomes by MALDI-quadrupole time-of-flight mass spectrometry and BLAST homology searching. Anal Chem. 73(9):1917-26.

Vogel H, Badapanda C, Knorr E, Vilcinskas A. 2014. RNA-sequencing analysis reveals abundant developmental stage-specific and immunityrelated genes in the pollen beetle Meligethes aeneus. Insect Mol Biol 23:98–112. <http://dx.doi.org/10.1111/imb.12067>.

Waterhouse RM, Seppey M, Simão FA, Manni M, Ioannidis P, Klioutchnikov G, Kriventseva EV, Zdobnov EM. 2017. BUSCO applications from quality assessments to gene prediction and phylogenomics. Mol Biol Evol 35, 543-548. doi: 10.1093/molbev/msx319

**Supplementary Figures**


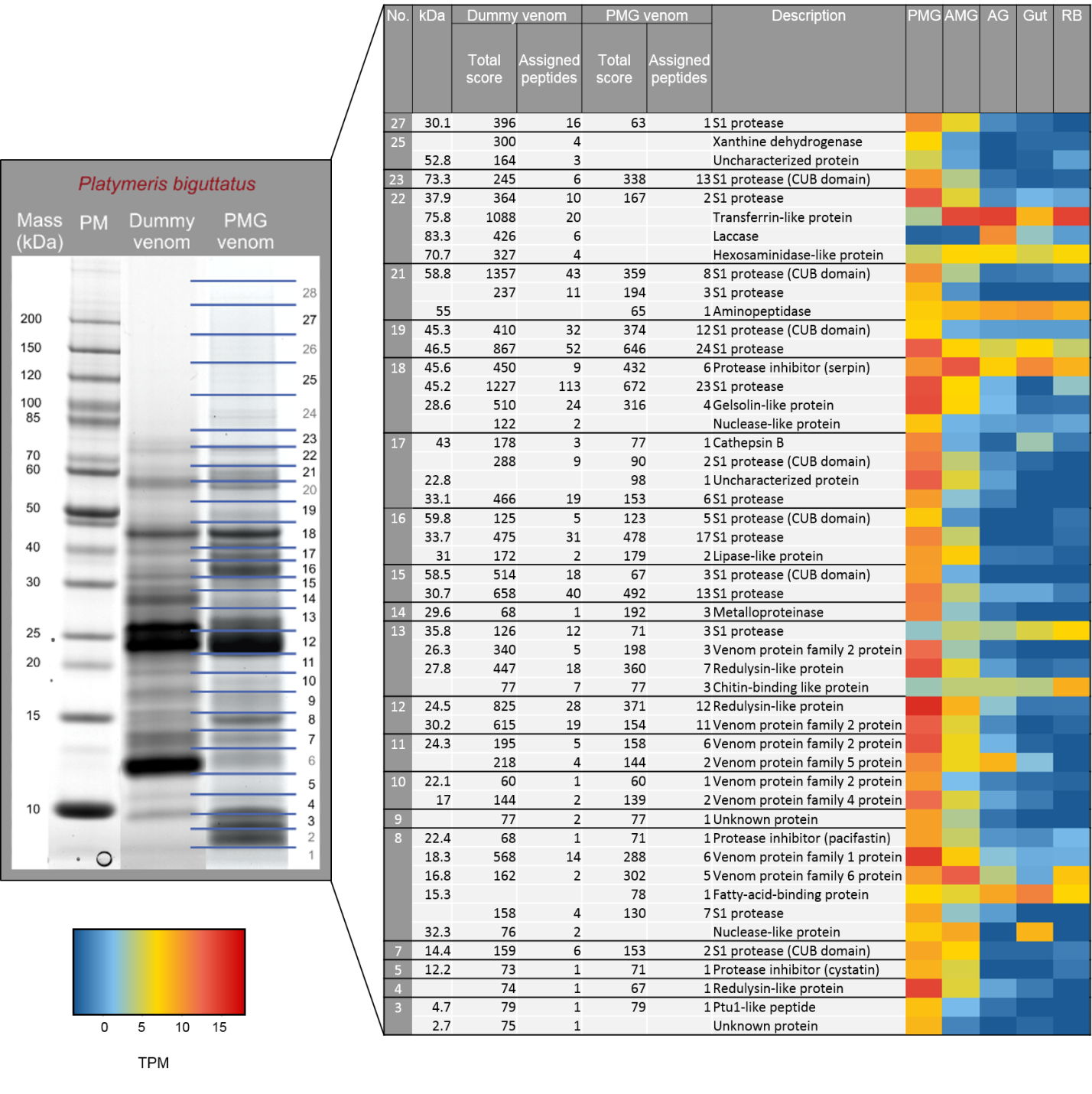


**Figure S1.** Proteins of the P. biguttatus PMG and prey dummy venom identified by LC-MS/MS. The Coomassie-stained protein gel on the left yielded the PMG venom proteins shown on the right, including the predicted protein masses (kDa), the total score, number of assigned peptides and descriptions. The excised bands are indicated with numbers and lines on the right side of the protein gel. For the proteins identified by LC-MS/MS, gene expression levels (log2 TPM) in the PMG, AMG, gut, and remaining body tissues are shown in the heat map. PM = protein marker. See Table S1 for the identity of matching predicted proteins in the P. biguttatus transcriptome.


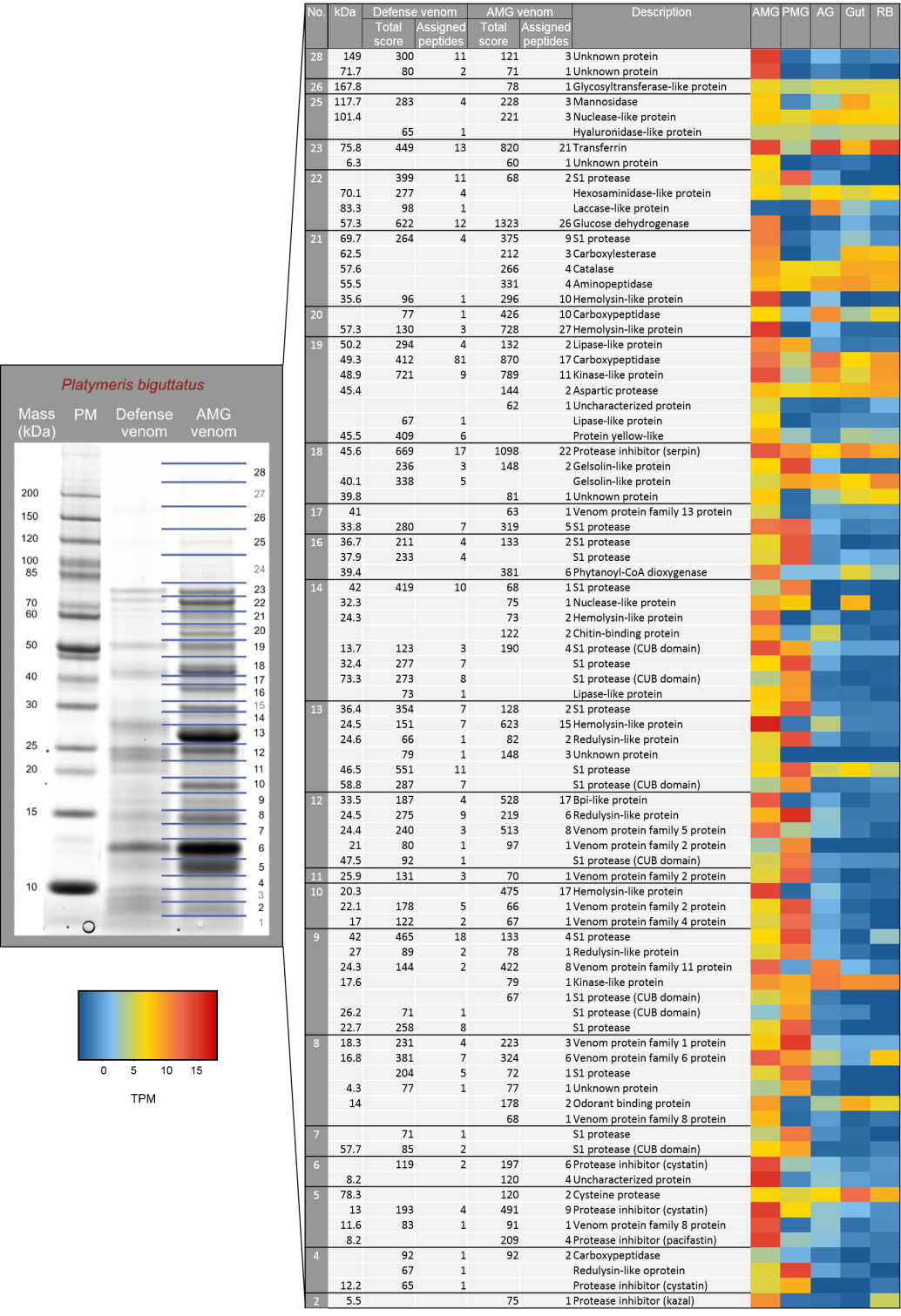


**Figure S2.** Proteins of the P. biguttatus AMG and defense venom (mild harassment) identified by LC-MS/MS. The Coomassie-stained protein gel on the left yielded the AMG venom proteins shown on the right, including the predicted protein masses (kDa), the total score, number of assigned peptides and descriptions. The excised bands are indicated with numbers and lines on the right side of the protein gel. For the proteins identified by LC-MS/MS, gene expression levels (log2 TPM) in AMG, PMG, gut, and remaining body tissues are shown in the heat map. PM = protein marker. See Table S1 for the identity of matching predicted proteins in the P. biguttatus transcriptome.
